# Elevated thymidine kinase 1 expression at baseline predicts poor prognosis in breast cancer patients

**DOI:** 10.3389/fonc.2026.1666576

**Published:** 2026-01-29

**Authors:** Peng Li, Yongting Cheng, Junfeng Zhao, Yuan Fang, Tingting Zhao

**Affiliations:** 1 Department of Medical Oncology, The First Affiliated Hospital of Hebei North University, Zhang jia kou, Hebei, China; 2 Department of Medical Laboratory Science, Hebei North University, Zhang jia kou, Hebei, China

**Keywords:** baseline TK1 expression, breast cancer, meta-analysis, OS, PFS, thymidine kinase 1

## Abstract

**Introduction:**

Thymidine kinase 1 (TK1), a key enzyme in DNA biosynthesis, has been shown to correlate with breast cancer prognosis and treatment response in dynamic monitoring settings. However, the clinical relevance of baseline TK1 levels remains controversial due to inconsistent evidence across studies. To address this issue, we conducted the first systematic meta-analysis of available studies to investigate the potential association between baseline TK1 levels and prognostic outcomes in breast cancer patients.

**Methods:**

A comprehensive computerized literature search was conducted across major Chinese and English databases to identify studies investigating the association between TK1 expression and breast cancer prognosis. Baseline TK1 expression levels and corresponding patient survival data were systematically extracted for meta-analysis.

**Results:**

The meta-analysis evaluating the association between baseline TK1 expression levels and progression-free survival (PFS) in breast cancer included 2,887 patients from 11 studies. Significant heterogeneity was observed across the included studies (*I*^2^ = 87.9%, *p* = 0.099), which persisted even after subgroup analyses. Therefore, a random-effects model was employed, yielding a pooled hazard ratio (HR) of 1.63 (95% confidence interval [CI]: 1.28–2.10, *p* = 0.000, *Z* = 3.88). The meta-analysis evaluating the association between baseline TK1 expression levels and OS in breast cancer included 2,233 patients from six studies. Significant heterogeneity was initially observed (*I*^2^ = 72.3%, *p* = 0.003), which was resolved through subgroup stratification by treatment status (treatment-naive versus recurrent disease). In the treatment-naive subgroup, the HR was 1.30 (95% CI: 1.11–1.52, *p* = 0.001, *Z* = 3.26). For the recurrent disease subgroup, the HR was 2.10 (95% CI: 1.74–2.54, *p* = 0.000, *Z* = 7.64).

**Conclusion:**

Breast cancer patients presenting with high baseline TK1 expression are associated with significantly worse prognostic outcomes. Collectively, these findings support the clinical potential of TK1 assessment for prognostic risk stratification and treatment guidance, which merits further verification in large-scale, multicenter clinical trials.

## 1 Introduction

Invasive breast cancer (IBC) is the most commonly diagnosed malignancy and the second leading cause of cancer-related mortality in women worldwide. Although standardized multimodal therapies have significantly improved disease-free survival (DFS) and overall survival (OS) in breast cancer patients, substantial interindividual variability in treatment response persists, driven by tumor biological heterogeneity and complex clinical factors (1). This heterogeneity underscores the critical need for robust prognostic biomarkers to enable precise risk stratification and personalized therapeutic decision-making.

Current clinical practice relies on established prognostic indicators, including hormone receptor status, human epidermal growth factor receptor 2 (HER2) expression, and Ki-67 proliferation index. The integration of comprehensive genomic profiling has further enhanced risk assessment and therapeutic selection, particularly for early-stage disease. However, despite these advancements, many proposed biomarkers exhibit inconsistent clinical utility and have not been widely adopted into routine clinical practice (2, 3).

A major clinical challenge remains the significant proportion of patients who experience disease recurrence or metastasis despite receiving optimal standard therapy, highlighting the urgent need for more accurate prognostic tools. The discovery of novel, reliable biomarkers could substantially improve risk prediction and facilitate the development of tailored treatment strategies.

In humans, thymidine kinase exists as two distinct isoenzymes: cytosolic thymidine kinase 1 (TK1) and mitochondrial thymidine kinase 2 (TK2) (4). As a cell cycle-regulated proliferation marker, TK1 is markedly upregulated during the *S*-phase to facilitate DNA synthesis. It plays a pivotal role in the pyrimidine salvage pathway—one of the two major routes for DNA precursor biosynthesis (alongside *de novo* synthesis)—thereby contributing to DNA replication and repair (5). Under normal physiological conditions, serum TK1 activity in healthy individuals is negligible or undetectable. By contrast, it is substantially elevated in malignant tumors, with strong correlations to tumor proliferation, progression, and metastatic potential (6). These findings have positioned TK1 as a promising biomarker for prognostic assessment and therapeutic decision-making in oncology.

While the utility of serial TK1 monitoring for treatment response and prognosis in breast cancer has been established, significant discrepancies persist regarding the diagnostic, prognostic, and predictive value of baseline TK1 levels (7–9). The correlation between baseline TK1 levels and distinct prognostic outcomes in breast cancer patients, as well as its potential utility in guiding diagnosis and treatment, remains controversial due to inconsistent conclusions across studies. To address this evidence gap, we conducted the first meta-analysis to systematically assess the relationship between baseline TK1 levels and prognosis in breast cancer.

## 2 Methods

### 2.1 Literature search

A comprehensive literature search was performed in both Chinese and international databases up to 31 May 2025. Chinese databases included China National Knowledge Infrastructure (CNKI), Weipu Information (VIP), Wanfang, and Chinese Biomedical Literature Database (CBMDisc), while English databases comprised PubMed (US National Library of Medicine), EMBASE, and the Cochrane Library. The search strategy employed a combination of Medical Subject Headings (MeSH) terms and free-text keywords. The following search terms were used: “thymidine kinase”, “thymidine kinase 1”, “TK”, or “TK1”, in combination with “breast cancer”. Both Chinese and English search terms were adapted to the respective databases to ensure comprehensive coverage.

### 2.2 Inclusion criteria

1. Original research articles published in peer-reviewed journals, with full text available in English or Chinese, focusing on primary breast cancer patients.
2. Studies investigating the association between TK1 expression levels and breast cancer prognosis, including OS, DFS, progression-free survival (PFS), or recurrence-free survival (RFS).
3. Clear definition of TK1 expression status, with patients stratified into high- and low-TK1 expression groups based on standardized thresholds (e.g., immunohistochemistry cutoff values, mRNA levels, or enzyme activity).
4. TK1 measurements obtained at initial diagnosis or after a treatment-free interval of ≥6 months, ensuring that TK1 levels were assessed prior to any therapeutic intervention (e.g., surgery, chemotherapy, or radiotherapy).
5. Reported survival outcomes (e.g., OS, DFS/PFS, RFS) with adjusted hazard ratios (HRs) and 95% confidence intervals (CIs) derived from multivariate Cox regression analyses (preferred) or univariate analyses if multivariate data were unavailable.
6. Sufficient statistical data to directly extract or calculate HRs and 95% CIs (e.g., Kaplan-Meier survival curves with log-rank P-values, event numbers, or regression coefficients).

### 2.3 Exclusion criteria

1. Non-English/non-Chinese articles; cross-sectional studies, experimental studies, review articles, conference abstracts, case reports, and letters.
2. Studies focusing on nonprimary breast cancer.
3. Studies lacking survival outcomes (e.g., OS, DFS) or with insufficient data to calculate/extract HRs with 95% CIs.
4. Studies unable to compare baseline TK1 levels (e.g., missing two-group HR data for high/low expression) or with insufficient data for meta-analysis pooling.
5. Studies with overlapping patient cohorts or duplicate data sources.

### 2.4 Information extraction

Two independent researchers systematically extracted data from eligible studies, with any discrepancies resolved through discussion or by consulting a third reviewer. Key extracted information included study characteristics (first author, publication year, country, design), patient demographics (sample size, age, stage), TK1 detection methods (specimen type, assay technique, cutoff values), and survival outcomes (HRs with 95% CIs for OS/DFS/PFS). For studies with multiple reports, the most comprehensive dataset was selected. Study quality was assessed using the Newcastle-Ottawa Scale (NOS), evaluating cohort selection, comparability, and outcome assessment. All extracted data were cross-verified to ensure accuracy prior to analysis.

### 2.5 Statistical analysis

Statistical analyses were performed using Stata 12.0 (StataCorp College Station, TX, United States), with HRs and 95% CIs for OS and PFS serving as the primary effect measures. Heterogeneity was assessed using *I*^2^ statistics and Cochran’s *Q* test (*p* < 0.10), with fixed-effects models applied when *I*^2^ < 50% and random-effects models applied when *I*^2^ ≥ 50%. For studies lacking reported HRs, estimates were derived from available survival data (10). Sensitivity analyses included sequential study exclusion and subgroup analyses. Publication bias was evaluated through funnel plots, Begg’s test, Egger’s test, and the trim-and-fill method. All tests were two-tailed, with statistical significance set at *p* < 0.05.

## 3 Results

### 3.1 Literature search

**Figure 1:**
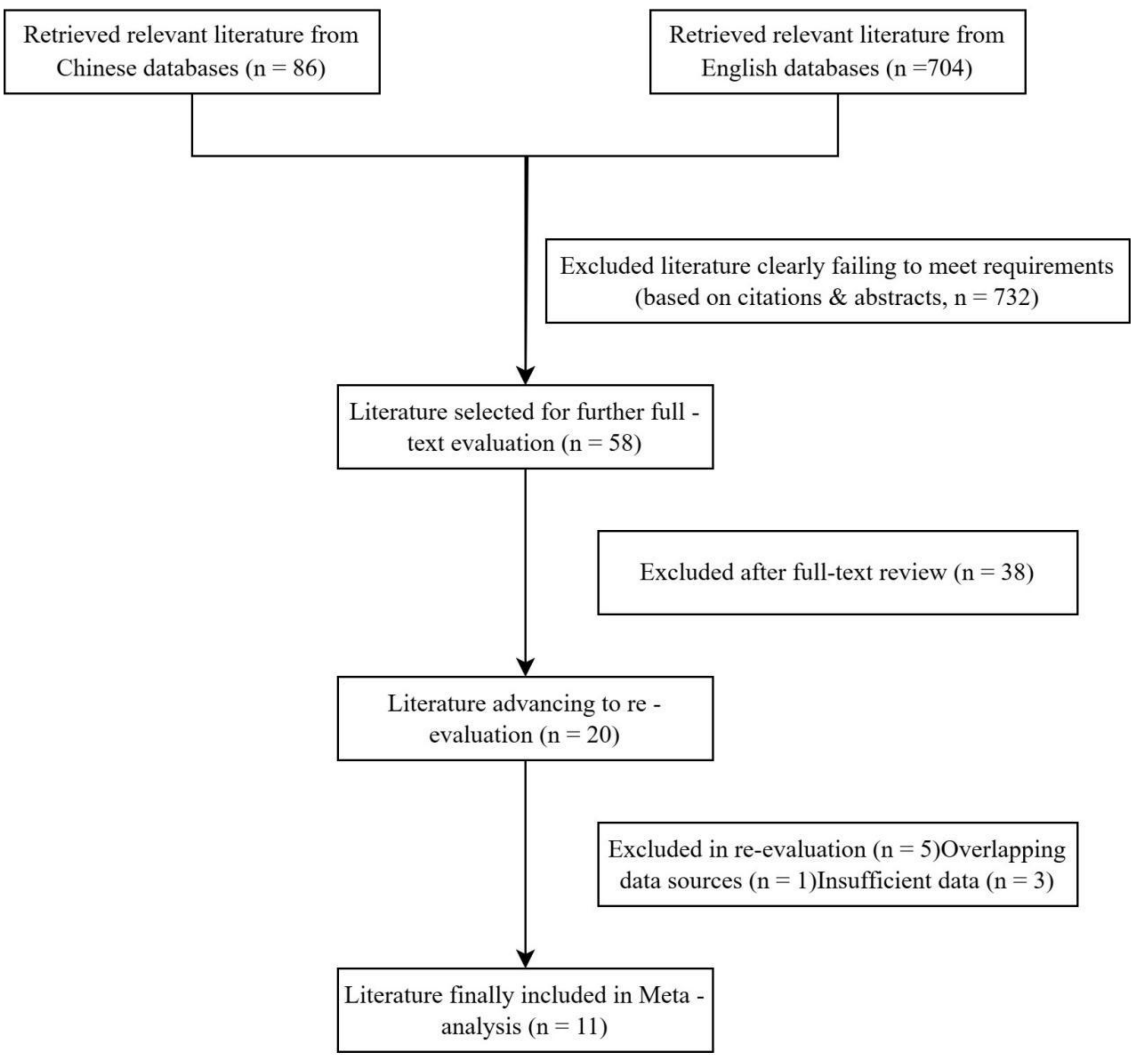
Literature identification and screening process.

The systematic literature search initially identified 790 potential studies from Chinese (CNKI, VIP, Wanfang, CBMDisc) and English (PubMed) databases. After removing 732 irrelevant records through title/abstract screening, 58 full-text articles were assessed for eligibility. Based on our predefined criteria, 47 studies were excluded, yielding 11 qualified studies for final meta-analysis (**Figure 1**).

### 3.2 Characteristics of included studies and literature quality evaluation

A total of 11 studies were included, with sample sizes ranging from 31 to 1,310. Based on the general disease characteristics of enrolled patients, five studies included primary treatment, while the remaining six included patients with recurrent disease. All 11 studies evaluated the correlation between TK1 levels and breast cancer prognosis. Although all reported PFS or DFS data comparisons, six also provided OS outcome comparisons. Geographically, four studies were conducted in Italy, four in Sweden, and the remaining three in France, the USA, and Israel. Regarding detection methods, nine studies used enzyme-linked immunosorbent assay (ELISA), one used immunohistochemistry, and one employed the bicinchoninic acid (BCA) method. Notably, six studies conducted multivariate analyses of factors associated with breast cancer prognosis. All eligible studies were identified from English-language databases and demonstrated high quality, as reflected in NOS scores of 8 to 9 (**Table 1**).

**Table 1:** Characteristics of included studies.

Author	Year	Region	Study design	Longest follow-up	Sample size	STT	DM	Cutoff	UA/MA	NOS
Broet (11)	2001	France	Retrospective analysis	203 months	1,310	Tumor tissue	BCA	Median value	MA	9
Nisman (12)	2010	Israel	Prospective study	More than 5 years	161	Serum	ELISA	> 134 DU/L	MA	9
Bjohle (13)	2013	Sweden	Retrospective analysis	Approximately 6 years	194	Serum	ELISA	≥ 235 Du/L (median)	MA	9
Bonechi (14)	2018	Italy	Retrospective analysis	More than 30 months	31	Serum	ELISA	≥ 122 Du/L (median)	UA	8
McCartney (15)	2019	Italy	Retrospective analysis	More than 8 months	221	Serum	ELISA	≥ 97 Du/L (median)	UA/MA	9
Larsson (16)	2020	Sweden	Prospective study	69 months	142	Serum	ELISA	≥ 391 Du/L (median)	UA/MA	9
McCartney (17)	2020	Italy	Retrospective analysis	More than 14 months	44	Serum	ELISA	≥ 318 Du/L (upper quartile)	UA	8
Fanelli (18)	2021	Italy	Retrospective analysis	More than 206 months	80	Tumor tissue	Immunohistochemistry	> 2.5%	UA	8
Matikas (9)	2021	Sweden	Retrospective analysis	More than 110 months	125	Serum	ELISA	Median value	MA	9
Bergqvist (19)	2023	USA/Canada	Retrospective analysis	More than 5 years	382	Serum	ELISA	≥ 250 Du/L (median)	UA	9
Zhu (20)	2024	Sweden	Prospective study	88 months	197	Serum	ELISA	Median value	UA	9

*STT*, specimen type for testing; *DM*, detection method; *RA*, retrospective analysis; *PS*, prospective study; *IHC*, immunohistochemistry; *ELISA*, enzyme-linked immunosorbent assay; *BCA*, bisquinoline carboxylic acid assay; *UA/MA*, univariate analysis/multivariate analysis; *NOS*, Newcastle-Ottawa Scale.

### 3.3 Meta-analysis of baseline TK1 expression and prognosis in breast cancer patients

**Figure 2:**
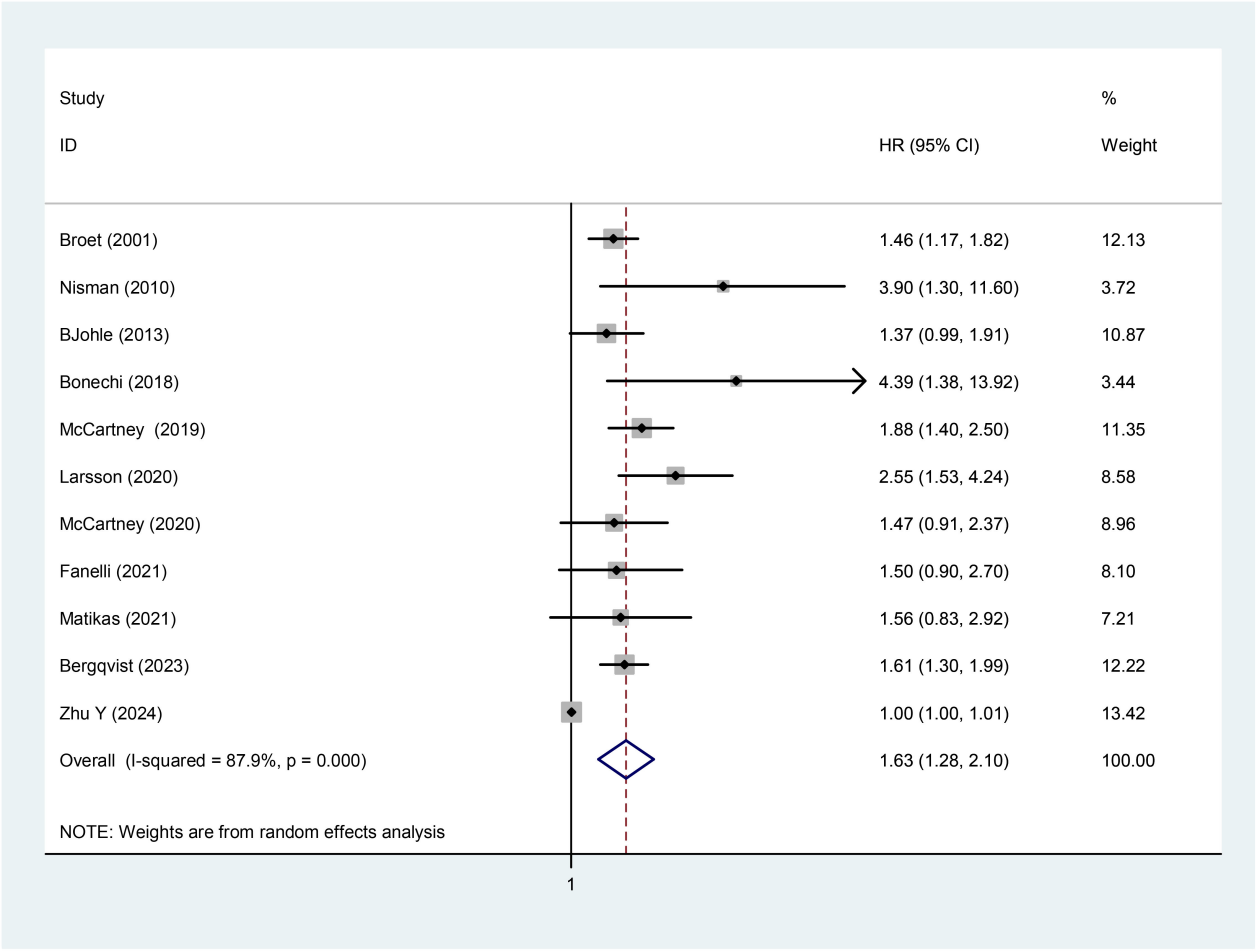
Meta-analysis of PFS in 11 studies (forest plot).

A meta-analysis was first performed to explore the correlation between baseline TK1 expression levels and PFS in breast cancer patients. For the PFS-focused studies (**Table 2**), heterogeneity testing revealed significant heterogeneity among the included studies (*I*^2^ = 87.9%, *p* = 0.099). A meta-regression analysis was conducted to assess the impact of treatment-naive/recurrent treatment status (as part of enrollment characteristics) on heterogeneity. The results showed that the treatment-naive/recurrent variable explained 34.03% of the heterogeneity, which was not statistically significant (Adj *R*-squared = 34.03%, *p* = 0.175). Considering that, in addition to treatment-naive/recurrent treatment status, baseline characteristics such as patient pathology and treatment modality across studies might also contribute to substantial heterogeneity—and that subgroup analyses could not fully eliminate this heterogeneity—a random-effects model was selected for the meta-analysis. The results indicated that baseline high TK1 expression predicted poorer PFS (HR: 1.63, 95% CI: 1.28–2.10, *p* < 0.001, *Z* = 3.88) (**Figure 2**).

**Table 2:** Association between baseline TK1 expression and PFS in breast cancer.

Author	Year	General characteristics of enrolled patients	Survival period	HR	LCI	UCI	*p-*value
Broet (11)	2001	Patients with primary operable unilateral nonmetastatic breast cancer	PFS	1.46	1.17	1.82	0.03
Nisman (12)	2010	Patients with primary operable invasive breast cancer without neoadjuvant chemotherapy	PFS	3.9	1.3	11.6	0.013
Bjohle (13)	2013	Patients with metastatic or locally inoperable advanced breast cancer who had not received treatment within 12 months before enrollment	PFS	1.37	0.99	1.91	0.059
Bonechi (14)	2018	Patients with metastatic breast cancer with hormone receptor-positive/HER2−	PFS	4.39	1.38	13.92	0.012
McCartney (15)	2019	Patients with hormone receptor-positive metastatic breast cancer	PFS	1.88	1.4	2.5	< 0.001
Larsson (16)	2020	Patients with metastatic breast cancer	PFS	2.55	1.53	4.24	< 0.001
McCartney (17)	2020	Postmenopausal patients with HR+/HER2− advanced breast cancer who had progressed on first- and second-line endocrine therapy (fulvestrant or AI monotherapy)	PFS	1.47	0.91	2.37	0.12
Fanelli (18)	2021	Patients with primary operable invasive breast cancer without neoadjuvant chemotherapy	PFS	1.5	0.9	2.7	0.13
Matikas (9)	2021	Patients with HER2-negative invasive breast cancer with a tumor diameter > 2 cm and primary operable	PFS	1.56	0.83	2.92	0.16
Bergqvist (19)	2023	Postmenopausal patients with HR-positive metastatic breast cancer who received endocrine therapy and had not received treatment within 12 months before enrollment	PFS	1.61	1.3	1.99	< 0.001
Zhu (20)	2024	Patients with HER2-positive primary breast cancer with a tumor size > 2 cm or confirmed lymph node metastasis	PFS	1.003	0.999	1.008	0.559

*HR*, hazard ratio; *LCI*, lower confidence interval; *UCI*, upper confidence interval; *PFS*, progression-free survival; *HER2*, human epidermal growth factor receptor 2; *HR-positive*, hormone receptor-positive; *AI*, aromatase inhibitor.

**Figure 3:**
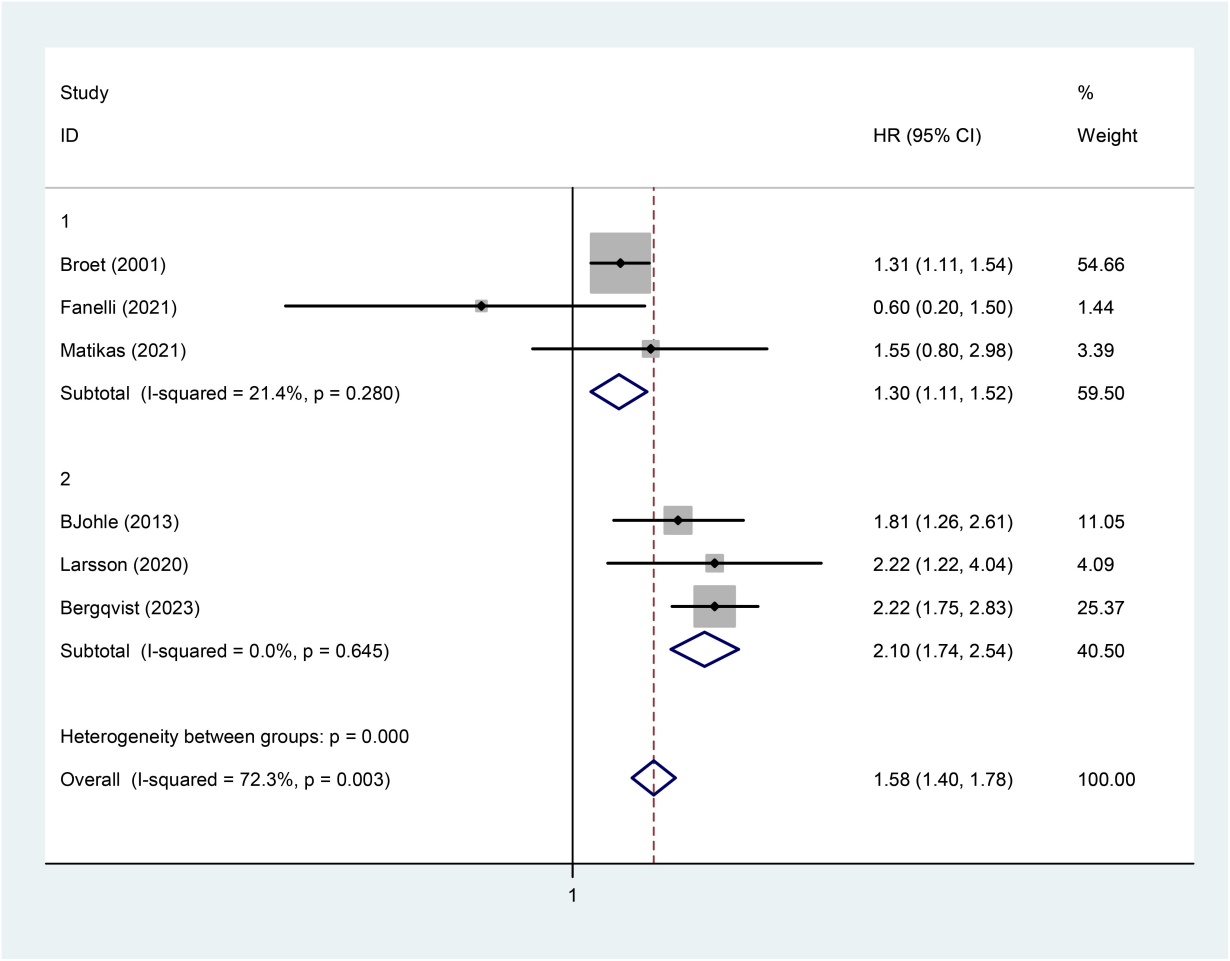
Meta-analysis of OS in six studies (forest plot).

A meta-analysis was performed to investigate the association between baseline TK1 expression and OS in breast cancer patients. For OS-focused studies (**Table 3**), heterogeneity testing revealed significant interstudy heterogeneity (*I*^2^ = 72.3%, *p* = 0.003). A meta-regression analysis was conducted to assess the impact of treatment-naive/recurrence status (as part of enrollment characteristics) on heterogeneity. The results showed that the treatment-naive/recurrence variable explained 100% of the heterogeneity, which was statistically significant (Adj *R*-squared = 100%, *p* = 0.019). Based on baseline treatment-naive/recurrence status, relevant studies were divided into two subgroups, and fixed-effects models were applied for separate meta-analyses. No significant heterogeneity was observed in either the treatment-naive subgroup (*I*^2^ = 21.4%, *p* = 0.28) or the recurrence subgroup (*I*^2^ = 0.0%, *p* = 0.645). The meta-analysis results indicated that baseline high TK1 expression predicted poorer OS in both subgroups (treatment-naive: HR = 1.30, 95% CI: 1.11–1.52, *p* = 0.001, *Z* = 3.26; recurrence: HR = 2.10, 95% CI: 1.74–2.54, *p* < 0.001, *Z* = 7.64) (**Figure 3**).

**Table 3:** Association between baseline TK1 expression and OS in breast cancer.

Author	Publication year	General characteristics of enrolled patients	Survival	HR	LCI	UCI	*p*-value
Broet (11)	2001	Patients with primary operable unilateral nonmetastatic breast cancer	OS	1.31	1.11	1.54	< 0.01
Bjohle (13)	2013	Patients with metastatic or locally inoperable advanced breast cancer who had not received treatment within 12 months before enrollment	OS	1.81	1.26	2.61	0.001
Larsson (16)	2020	Patients with metastatic breast cancer scheduled to receive first-line treatment	OS	2.22	1.22	4.04	0.009
Fanelli (18)	2021	Patients with primary operable invasive breast cancer without neoadjuvant chemotherapy	OS	0.6	0.2	1.5	0.27
Matikas (9)	2021	Patients with HER2-negative invasive breast cancer with a tumor diameter > 2 cm and primary operable	OS	1.55	0.8	2.98	0.19
Bergqvist (19)	2023	Postmenopausal patients with HR-positive metastatic breast cancer who received endocrine therapy and had not received treatment within 12 months before enrollment	OS	2.22	1.75	2.83	< 0.001

*HR*, hazard ratio; *LCI*, lower confidence interval; *UCI*, upper confidence interval; *OS*, overall survival; *HER2*, human epidermal growth factor receptor 2; *HR-positive*, hormone receptor-positive.

### 3.4 Sensitivity analysis

**Figure 4:**
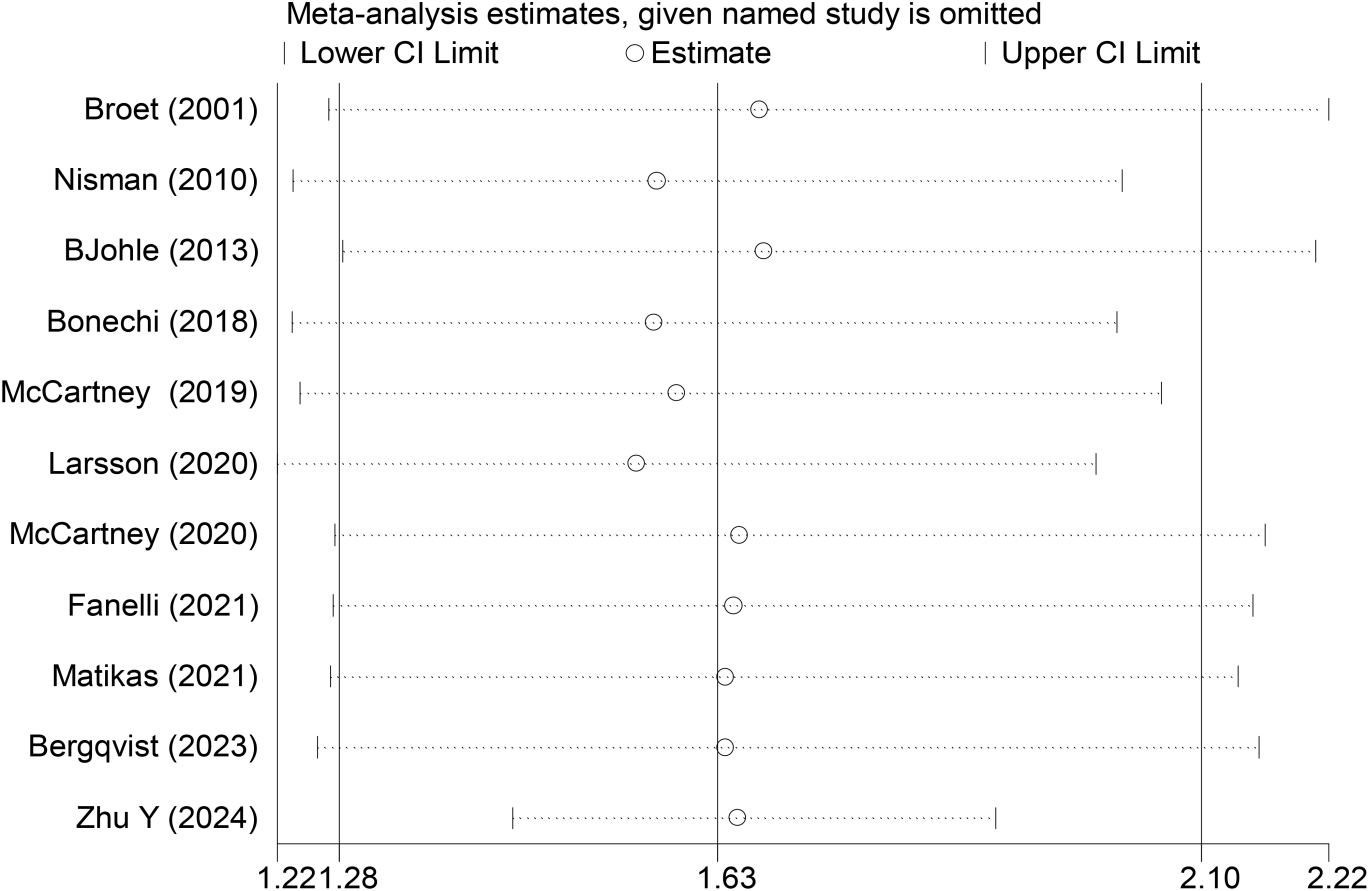
Sensitivity analysis of PFS data.

In this study, sensitivity analyses were performed using leave-one-out exclusion to evaluate the robustness of the meta-analysis results. For PFS-related studies, sequential exclusion of each included study showed that the direction of pooled effect sizes in the remaining studies remained consistent, and the 95% CIs of the pooled effect sizes did not include the null value of 1.0. These results indicated that the absence of any individual study did not significantly alter the overall conclusions, suggesting that the meta-analysis findings were not substantially influenced by single studies (**Figure 4**).

**Figure 5:**
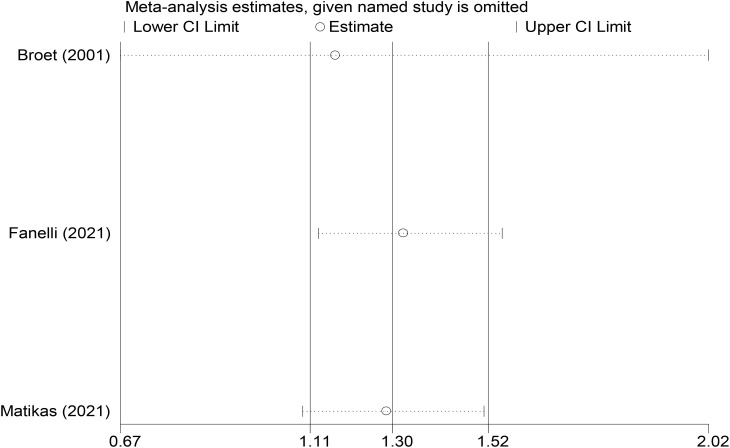
Sensitivity analysis of OS in treatment-naive subgroups.

In the sensitivity analysis of the OS treatment-naive subgroup, exclusion of the 2001 Broet study—which enrolled a patient cohort with a notably distinct treatment background compared to those in subsequent investigations—resulted in the 95% CIs of the HR encompassing 1.0. Furthermore, whereas TK1 was measured in tumor tissue in this study, the majority of subsequent investigations utilized serum samples. This biological discrepancy in sample sources may introduce systematic bias when comparing the prognostic utility of TK1 across these studies. Combined with the sensitivity analysis results and the observations mentioned above, this indicates that the Broet study critically influenced the statistical significance of the overall effect, reflecting the poor stability of the pooled effect size (**Figure 5**).

**Figure 6:**
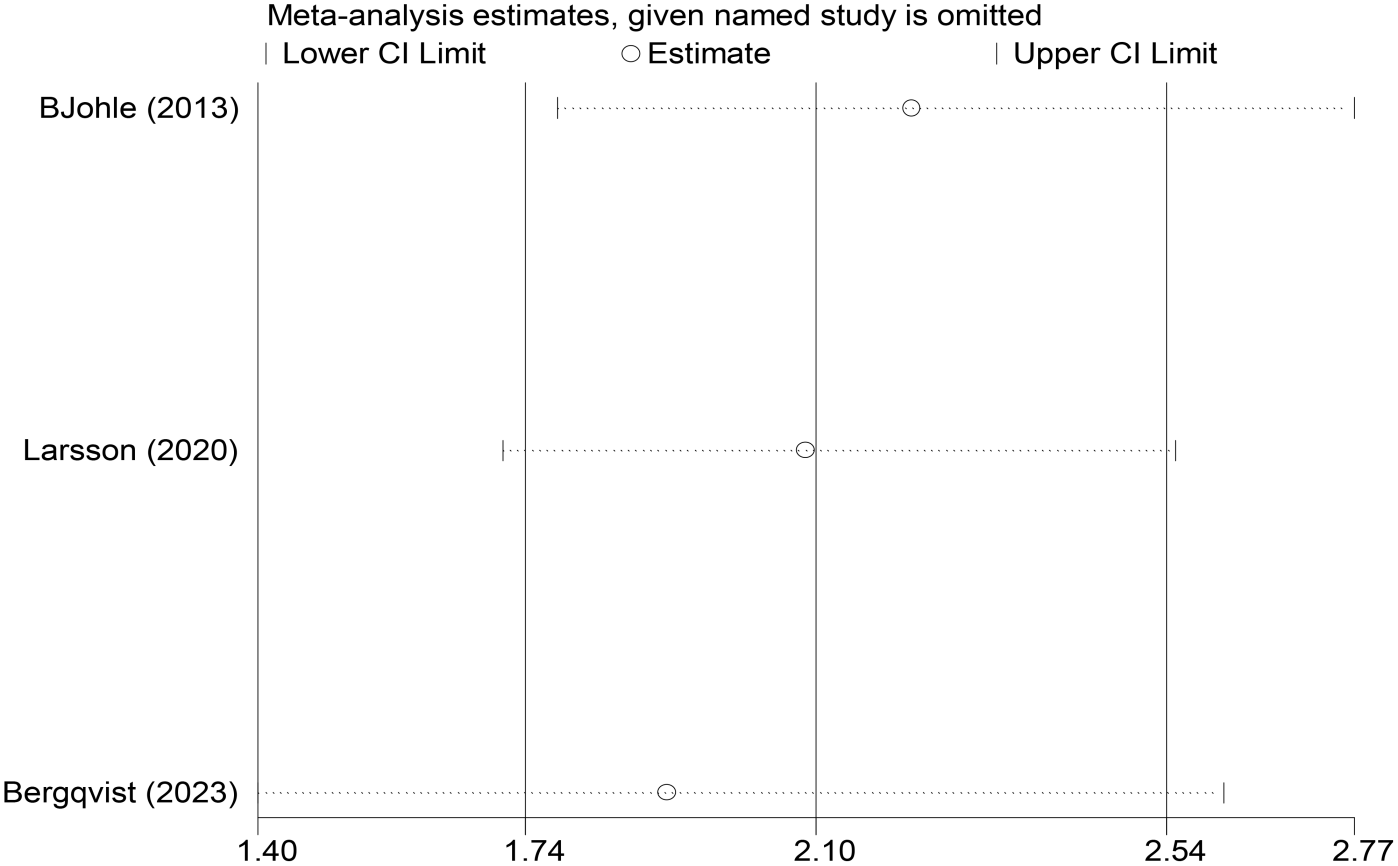
Sensitivity analysis of OS in recurrence subgroups.

In the sensitivity analysis of OS recurrence subgroup studies, sequential exclusion of each included study showed that the direction of pooled effect sizes in the remaining studies remained consistent, and none of the 95% CIs for the pooled effect sizes included the null value of 1.0. These results indicated that the absence of any individual study did not significantly alter the overall conclusions, suggesting that the meta-analysis findings were not substantially influenced by single studies (**Figure 6**).

### 3.5 Publication bias

**Figure 7:**
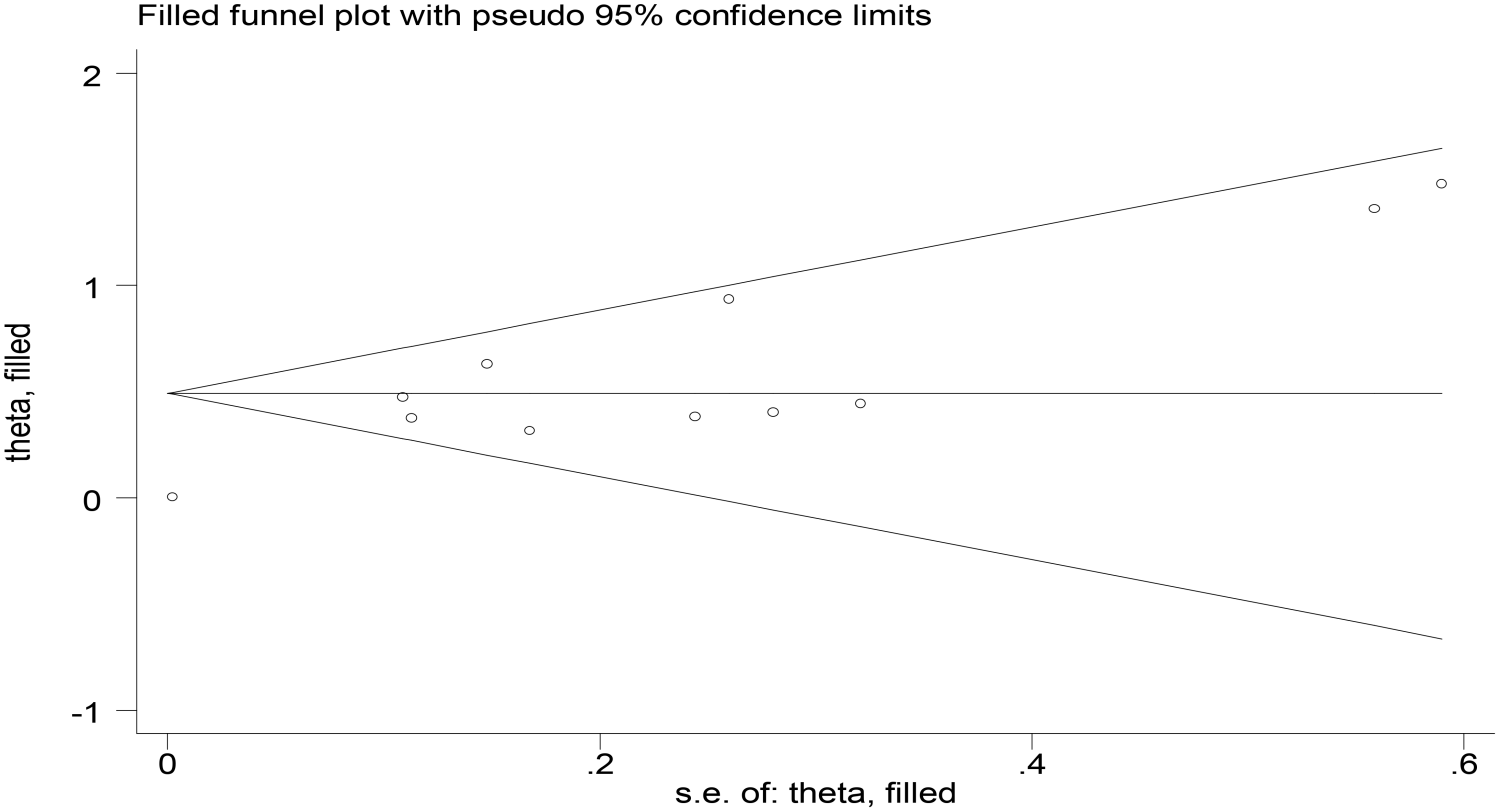
Funnel plot assessing publication bias in PFS studies (posttrim-and-fill adjustment).

For the meta-analysis of baseline TK1 expression and PFS involving 11 studies, publication bias was assessed using Begg’s and Egger’s tests. The results showed no significant publication bias (*p* > 0.05). Additionally, the trim-and-fill method detected no studies requiring trimming or filling. Collectively, these findings indicate that the included studies were not substantially affected by publication bias (**Figure 7**).

**Figure 8:**
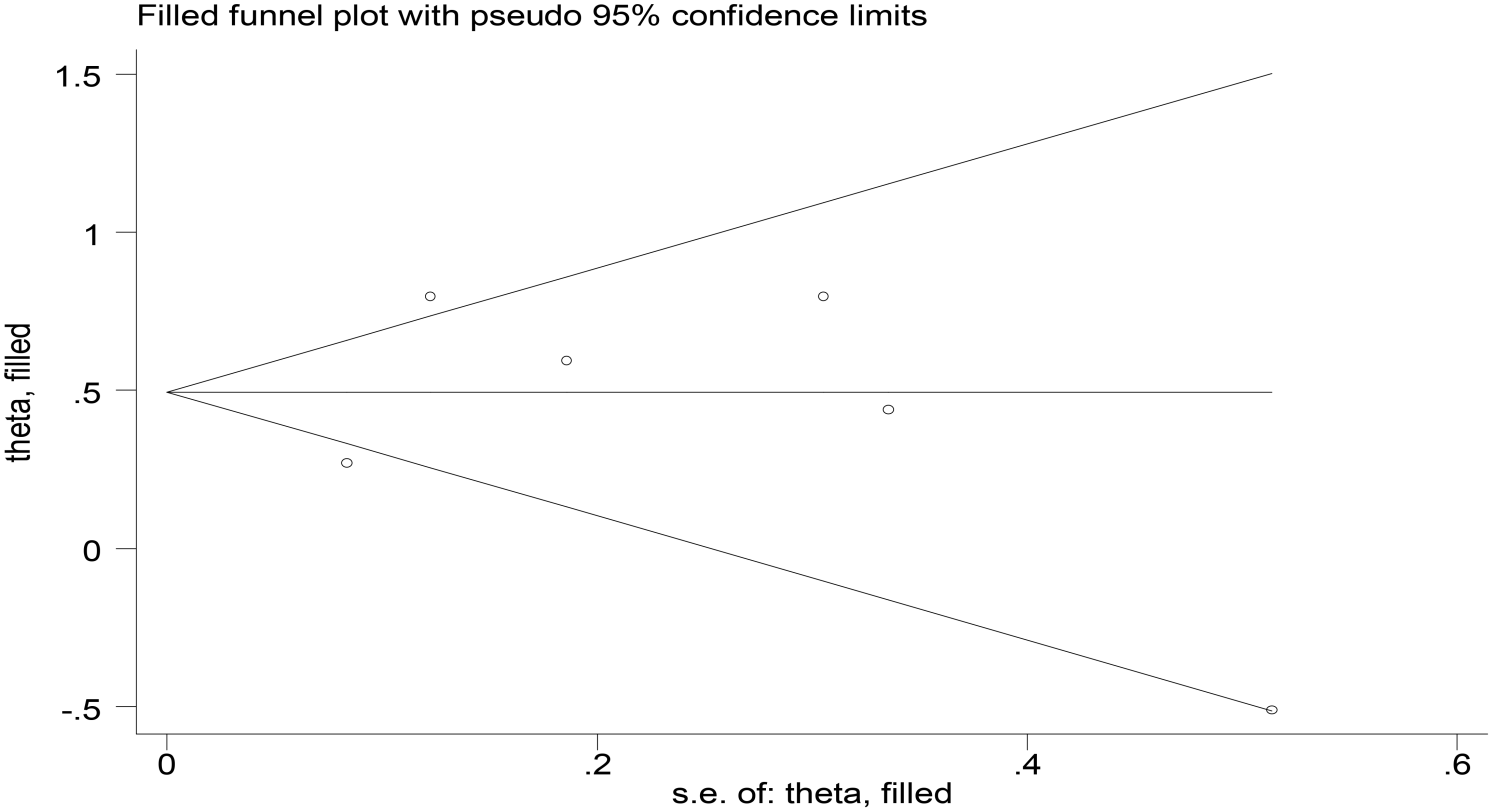
Funnel plot assessing publication bias in OS studies (posttrim-and-fill adjustment).

Publication bias was assessed for the six studies included in the meta-analysis of baseline TK1 expression and OS. Results from Begg’s and Egger’s tests indicated no significant publication bias (*p* > 0.05). The trim-and-fill method also detected no studies requiring adjustment. Given the limited number of studies included in the OS analysis, the statistical tests used to detect publication bias may be underpowered. Therefore, the potential for publication bias cannot be entirely ruled out (**Figure 8**).

## 4 Discussion

This meta-analysis evaluated the prognostic significance of baseline TK1 expression in breast cancer, with strict inclusion criteria requiring TK1 assessment prior to treatment initiation. Pooled analysis of 11 high-quality studies (NOS scores 8–9) encompassing diverse clinical subtypes (early/advanced stage, various receptor statuses, and operable/inoperable cases) revealed significantly worse progression-free survival in patients with elevated baseline TK1 (HR = 1.63, 95% CI: 1.28–2.10, *p* < 0.001; random-effects model, *I*^2^ = 87.9%).

The significant heterogeneity observed may originate from several potential sources. First, clinical heterogeneity across patient populations likely contributed substantially. The included studies involved a broad spectrum of breast cancer patients, ranging from those with early-stage operable disease (9, 11, 18) to those with advanced metastatic disease (13–17, 19), as well as patients receiving neoadjuvant therapy (9, 20). Notable differences exist between early- and advanced-stage patients regarding tumor burden and the tumor microenvironment. For example, TK1 may more directly reflect high proliferative activity and disease burden in advanced-stage patients, whereas in early-stage patients, it could be associated with micrometastatic potential. Although a subgroup analysis based on treatment-naive versus recurrent disease was performed, this stratification did not fully account for the observed heterogeneity(Adj *R*-squared = 34.03%, *p* = 0.175), suggesting that additional clinicopathological factors—such as hormone receptor status, HER-2 expression, prior lines of therapy, and metastatic sites—may also influence the results.

Second, methodological variations in detection assays and specimen types represent another key source of heterogeneity. As summarized in **Table 1**, most studies (nine of 11) measured serum TK1 levels using ELISA. However, considerable differences existed among studies in the commercial kits used and in the definitions of positive thresholds (e.g., median, upper quartile, or prespecified cutoff values). Moreover, one study employed immunohistochemistry (IHC) to assess TK1 protein expression in tumor tissue (18), whereas an earlier study utilized the BCA method (11). Importantly, IHC captures *in situ* TK1 expression within tumor tissue, whereas ELISA quantifies circulating TK1 in peripheral blood. These two approaches reflect biologically distinct dimensions of TK1, differing in quantitative units, analytical sensitivity, and clinical interpretation—differences that inevitably introduce substantial heterogeneity across studies. Due to the limited overall number of available studies, performing univariate subgroup analyses for heterogeneity factors beyond treatment−naive/recurrent status would further reduce the number of studies in each subgroup, thereby precluding a meaningful examination or reduction of heterogeneity through subgroup comparisons. For these reasons, a random−effects model was ultimately employed to conduct the meta−analysis of PFS.

Analysis of six studies reporting overall survival demonstrated consistent prognostic value across subgroups (treatment-naive: HR = 1.30; recurrence: HR = 2.10; pooled HR = 1.58, 95% CI: 1.40–1.78, *p* = 0.003), with heterogeneity resolved through stratification by disease status. Based on the observed disparity in HR between the two subgroups, the prognostic significance of TK1 appears to be disease stage-dependent. These findings confirm that baseline TK1 expression is consistently associated with adverse outcomes in breast cancer patients, establishing it as a reliable prognostic indicator.

As a key enzyme in the DNA salvage pathway, TK1 demonstrates peak expression during the S-phase of the cell cycle, serving as a precise indicator of tumor proliferative activity. Its biological properties have been well characterized. Current research on TK1 spans diverse solid and hematological malignancies (21–24), with the most clinically significant positive findings concentrated in breast cancer (25). Accumulating evidence has established TK1 as a key mediator of breast cancer progression. Recent mechanistic studies have further defined its specific function as a downstream effector of the cyclin-dependent kinase 4/6 (CDK4/6) pathway. In patients with hormone receptor-positive/human epidermal growth factor receptor 2-negative (HR+/HER2−) advanced breast cancer, an early elevation in TK1 activity following CDK4/6 inhibitor administration is consistently correlated with decreased endocrine therapy sensitivity or the development of acquired drug resistance (26, 27). Our findings substantiate the clinical utility of baseline TK1 levels in prognostic assessment for breast cancer. Importantly, baseline TK1 maintained stable prognostic performance across diverse clinical subtypes, suggesting its potential to overcome the limitations of conventional pathological classification and provide more broadly applicable clinical guidance.

Nevertheless, this study has several limitations that should be acknowledged. The primary limitation stems from substantial heterogeneity in the progression-free survival analysis (*I*^2^ = 87.9%), which persisted despite employing disease status stratification and other statistical adjustments. Interpretation of PFS outcomes from this meta-analysis requires caution, as the high heterogeneity observed suggests that the prognostic value of TK1 may be influenced by variations in patient characteristics and detection methodologies. To consolidate the clinical utility of TK1, subsequent investigations are recommended to establish standardized inclusion criteria and uniform TK1 detection protocols, coupled with rigorous prospective validation studies, thereby yielding robust evidence to advance the development and optimization of TK1-driven therapeutic strategies. Second, our analysis may be susceptible to publication bias, in which studies demonstrating a significant association between TK1 expression and poor prognosis are more likely to be published, potentially leading to an overestimation of its true prognostic value. Although an exhaustive literature search was conducted, this limitation cannot be entirely excluded.

Building on our findings, we propose three critical directions for future investigation: (1) establishing standardized TK1 detection protocols to enhance interlaboratory reproducibility and clinical applicability; (2) conducting mechanistic studies to elucidate TK1’s prognostic role in breast cancer, with particular emphasis on its crosstalk with core signaling pathways to inform targeted therapy development; and (3) performing biomarker-driven clinical trials to evaluate TK1-guided therapeutic strategies, especially in patients exhibiting elevated TK1 expression.

An optimal prognostic biomarker should fulfill a tripartite role: outcome prediction, treatment response monitoring, and guidance for therapeutic decision-making. Our meta-analysis demonstrates a significant association between elevated baseline TK1 expression and adverse prognostic outcomes in breast cancer. Although high baseline TK1 expression indicates poor prognosis, whether adjusting treatment strategies in advance or implementing more active interventions can improve clinical benefits for these patients under the current treatment system requires further support from high-quality clinical research data.

## Data Availability

The raw data supporting the conclusions of this article will be made available by the authors, without undue reservation.

## Supplementary material

Supplementary Table 1 Detailed quality assessment of included cohort studies using the Newcastle-Ottawa Scale (NOS). Scoring Guide: ★: One star awarded for fulfilling the criterion. Selection (S): S1:​ Representativeness of the Exposed Cohort. S2:​ Selection of the Non-Exposed Cohort. S3:​ Ascertainment of Exposure (e.g., TK1 measurement method). S4:​ Demonstration that outcome of interest was not present at start. Comparability (C): C1:​ Comparability of cohorts on the basis of the design or analysis (controlling for confounders). The most important factor(s) controlled for are noted (e.g., age, tumor stage). Outcome (O): O1:​ Assessment of Outcome (e.g., progression, death). O2:​ Was follow-up long enough for outcomes to occur? O3:​ Adequacy of follow-up of cohorts.
